# Effect of predictive sign of acceleration on heart rate variability in passive translation situation: preliminary evidence using visual and vestibular stimuli in VR environment

**DOI:** 10.1186/1743-0003-4-36

**Published:** 2007-09-29

**Authors:** Hiroshi Watanabe, Wataru Teramoto, Hiroyuki Umemura

**Affiliations:** 1 Institute for Human Science and Biomedical Engineering, National Institute of Advanced Industrial Science and Technology, Ikeda, Osaka, Japan

## Abstract

**Objective:**

We studied the effects of the presentation of a visual sign that warned subjects of acceleration around the yaw and pitch axes in virtual reality (VR) on their heart rate variability.

**Methods:**

Synchronization of the immersive virtual reality equipment (CAVE) and motion base system generated a driving scene and provided subjects with dynamic and wide-ranging depth information and vestibular input. The heart rate variability of 21 subjects was measured while the subjects observed a simulated driving scene for 16 minutes under three different conditions.

**Results:**

When the predictive sign of the acceleration appeared 3500 ms before the acceleration, the index of the activity of the autonomic nervous system (low/high frequency ratio; LF/HF ratio) of subjects did not change much, whereas when no sign appeared the LF/HF ratio increased over the observation time. When the predictive sign of the acceleration appeared 750 ms before the acceleration, no systematic change occurred.

**Conclusion:**

The visual sign which informed subjects of the acceleration affected the activity of the autonomic nervous system when it appeared long enough before the acceleration. Also, our results showed the importance of the interval between the sign and the event and the relationship between the gradual representation of events and their quantity.

## Background

Recent advances in video display technology have produced large, high-definition displays that can produce a strong sense of the viewer's own motion (vection) with only visual input that lacks any vestibular input. This dynamic environment differs from real-world experiences in which various senses are stimulated simultaneously. We therefore believe that investigation of the effects of such an environment on the human is important for establishing a safe video presentation environment. In the real world, too, recent growth in transportation facilities is presenting us with a great increase in opportunities to ride in vehicles as passive passengers. It also means that passengers will more often be subjected to high speeds and extraordinary accelerations over long periods of time. We therefore believe that the development of technology for reducing the psychological load on travelers is important to maintaining comfort in public transportation. These two conditions, vection from exposure to moving images and riding as a passenger, share the common point of the body being passively subjected to motion. Passive movement differs from active movement in the control over the means of motion is not initiated in the brain of the passenger. However, Griffin [1] has shown that observers who cannot control their own movement, such as passengers in a vehicle, mainly attempt to use visual information to predict their motion. Naturally, the prediction often fails, and the contradictions in sensory feed-back that often occur at such times may cause feelings of discomfort. We can therefore expect that the psychological burden on the passenger could be suppressed by providing information that would supplement the prediction process. This approach has led to a number of reports concerning the effectiveness of 'prediction' with respect to the effects on the human body of virtual reality (VR) scenes experienced passively. Lin et al. [2] used a combined motion base mechanism and immersive VR system to present test subjects with transportation scenery. The scenery was presented in two ways. In one, visual points followed the course of the road; in the other, the visual points moved independently of the road. They reported that the observers' prediction of their own motion from the road course affected the comfort of their VR experience. In addition, [3] and [4] have attempted to reduce the discomfort produced by a VR environment by continuously providing a guide stimulus that draws attention to the direction of motion in a 3D VR space. The work we report here broadly follows the previous research paradigm, but our objective is to verify the possibility of affecting the activation state of the autonomic nervous system by presenting predictive information only when acceleration will occur, rather than constantly signaling the direction of movement to the observer.

There have been many proposals to quantitatively measure the effects of VR content using physiological indices such as the electrocardiogram, electrogastrograph, and galvanic skin response. The advantages of using physiological indices to understand the physical condition include a relatively light burden on the test subject during measurement and the ability to detect fluctuations over small time intervals. Relations between such indices and responses to dynamic environments have been reported in recent years (lowering of body temperature [5], visually-induced instability of center of gravity and activity of the autonomic nervous system [6], nausea and autonomic nervous system activity caused by excessive camera movement in motion pictures [7], and vestibular Coriolis stimulation [8]). In particular, results on the relation of a frequency analysis of changes in heart rate obtained from an electrocardiogram to autonomic nervous system activity have been pointed out since the 1980s, and there have been previous attempts to quantitatively measure the autonomic nervous system activity of test subjects in a dynamic environment [7,9-11]. The original approach to the relation between change in heart rate and autonomic nervous system activity was proposed by Akselrod et al. [12], who suggested that the low-frequency component of the change in heart rate reflects the activation state of both the sympathetic nervous system and parasympathetic (vagal) nervous system, and the high-frequency component reflects the activity of only the parasympathetic nervous system. Furthermore, the activity levels of the sympathetic nervous system and the parasympathetic nervous system exhibit a trade-off relationship, so the possibility of estimating the state of sympathetic nervous system activity by calculating the power ratio of the high-frequency and low-frequency components was considered. A relation between autonomic nervous system activity and psychological load has been suggested, and the use of that measure as an index for psychological load in a VR environment has been proposed a number of times in previous research. Of course there are many difficulties involved in determining the correspondence between this index and intrinsic mental states, but it is believed that there has been sufficient discussion on combining the data with questionnaire results to produce time-series data on inner states [13].

We measured the heart rate of test subjects as a time series while they were experiencing a driving simulator that combined a motion base and an immersive VR system. We used the heart rate to infer the activity state of the autonomic nervous system. Our main objective was to elucidate the effect of the presence or absence of a visual sign that predicts the direction of movement on the activity of the autonomic nervous system. The psychological and physiological states of the test subjects under conditions that produce VR sickness or motion sickness are outside the scope of this research. We created VR content free of movement information that produces conflict between the vestibular system and the visual system and presented the content to the test subjects in an environment in which VR sickness is not expected to occur. This procedure is designed to investigate the relation between signs that predict the direction of movement and the activity of the autonomic nervous system in a situation that approximates an actual motion scenario as closely as possible.

## Methods

### Subjects

Twenty-two university students participated in the experiments as paid volunteers, and none of them knew about the hypothesis of this study. Nine male subjects (23.8 ± 1.9 years) and 13 female subjects (24.8 ± 3.0 years) were used. Subjects recruited for this study underwent visual, vestibular, auditory, and cognitive screenings for undiagnosed problems that would prevent them from completing the study. We also asked subjects to fill out a questionnaire concerning physical condition and motion sickness prior to the experiments. The five questions on the questionnaire were, 1) Do you have a hangover?, 2) Did you have enough sleep last night?, 3) How often do you drive? (every day, sometimes, never), 4) How often do you experience car sickness? (often, sometimes, often in childhood, never), and 5) Have you ever seen a doctor for dizziness? Our study was approved by the Research Ethics Committee of the National Institute of Advanced Industrial Science and Technology, and the experiments were undertaken with the informed written consent of each subject.

### Apparatus

#### CAVE

**Figure 1:**
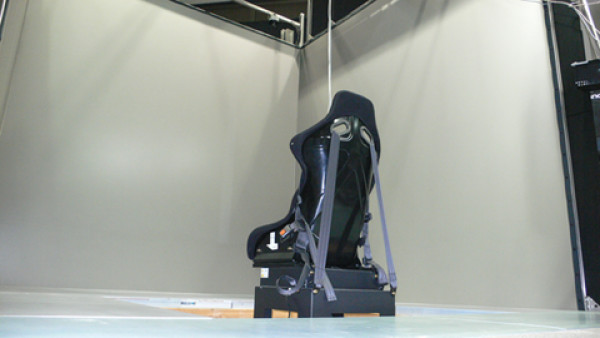
**CAVE and motion base system**. An immersive virtual reality system with four screens (front, floor, and two sides: 3 × 3 m) provided the subjects with monolithic stereoscopic graphics crossing multiple screens. The motion base system set up under the floor of CAVE made vestibular stimuli and synchronized them with the display.

A 3D display was presented by an immersive virtual reality system (CAVE, EVL at the University of Illinois, Figure 1). This system consisted of four 3 × 3 m screens (front, floor, and two sides) and stereo displays were projected on these screens at a 40-Hz refresh rate. Subjects observed the 3D display wearing polarized glasses, and the projection of the display was adjusted to their head position using a head tracker mounted on the glasses at a 1000-Hz sampling rate. A graphics workstation (Onyx/Infinite Reality, Silicon Graphics Inc.) generated the graphics display and controlled the motion base unit (see next section). Using this kind of immersive virtual reality system makes it possible to present a 3D visual field that includes almost the entire front, left, right, and ground surface visual fields. The optical flow with respect to the peripheral vision in particular can provide the subject with a strong sense of his or her own motion (vection) [14,15]. We can therefore expect to provide the subject with a stronger moving scenery simulation.

#### Motion Base System

Vestibular information synchronized with the display was generated by the motion base system (Mitsubishi Precision Inc.). This system was set up under the floor of CAVE (Figure 1) and could provide arbitrary rotation around three axes with six electromotive actuators (maximum angle of rotation: ± 12° around yaw, pitch, and roll). The acceleration and deceleration for the forward direction during driving were represented by the transformed angle around the pitch axis, and rotation in the driving plane was represented by the transformed angle around the yaw axis.

#### Data Analysis

In this section we described the method of electrocardiogram recording and defined the index of activity of the autonomic nervous system and, finally, summarized the relationship between the index and the activity of the autonomic nervous system that was introduced in previous studies.

**Figure 2:**
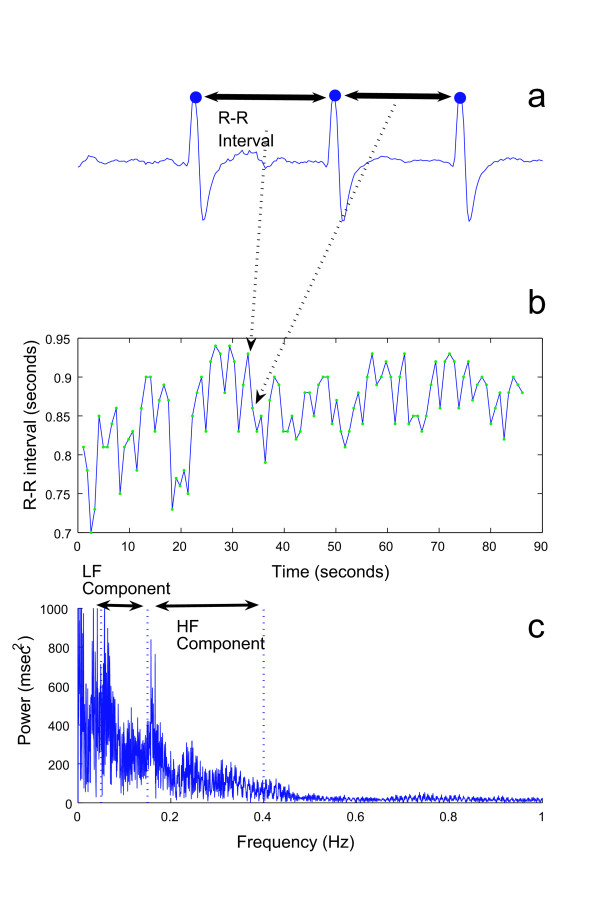
Example of ECG data and R wave (a), R-R trendgram (b), and FFT results from R-R trendgram (c).

An electrocardiogram (ECG) was measured from a pair of Ag-AgCl electrodes placed on the chests of subjects (MP150, BIOPAC Systems Inc.). All analog signals were amplified and digitized at a 1-kHz sampling rate (12-bit resolution) using a telemeter (SYNACT-MT11, Nihondenki Inc.) and stored on a hard disk for later analysis (PC-MA10TEZE65J9, NEC). We first detected the peaks, R waves, from approximately sixteen minutes of ECG data. We then calculated the time interval from one R wave to the next (Figure 2a). The set of these intervals, which we called "R-R intervals," shows the heart rate variability (HRV) (Figure 2b). Many previous studies have suggested that the spectral parameters derived from FFT algorithm applied to HRV relate to the activity of the autonomic nervous system based on an antagonistic function between the sympathetic and parasympathetic nervous systems. In the frequency domain, HRV often has two principle spectral components. The low frequency (LF) component (0.05–0.15 Hz) is linked to the sympathetic modulation, but also includes some parasympathetic influence; the high frequency (HF) spectral band (0.15–0.4 Hz) reflects parasympathetic activity [16,17] (Figure 2c). Thus, the ratio of the LF and HF spectral components (the LF/HF ratio) is an index of the activity ratio of the sympathetic and parasympathetic nervous systems; a high value means the dominance of the sympathetic system and a low value means the dominance of the parasympathetic system.

### Procedure

#### Simulated Driving Course

The virtual driving course that is presented to the subject was created with the following constraints. Changes in forward velocity consist of a recurring block of four events: acceleration, constant velocity, deceleration, constant velocity. The time intervals for the four events are 28.5 ± 5.7 seconds. The constant velocity is determined at random in the range from 10 km/h to 70 km/h. Turning events occur every 23.5 ± 4.7 seconds, selected randomly in the range of plus or minus 4.5 degrees. The turning direction, left or right, is reversed for each turning event. There is no relation between the acceleration or deceleration events and the turning events.

**Figure 3:**
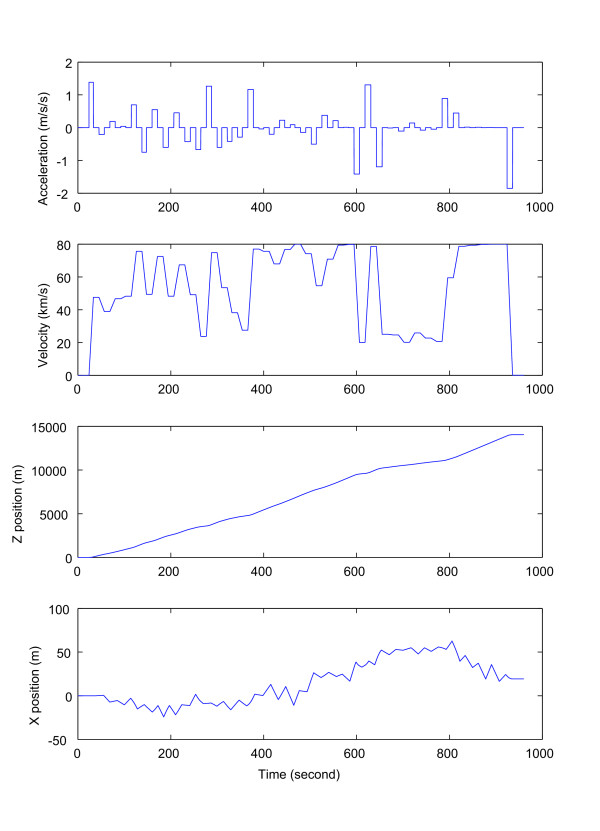
**Simulated driving schedule**. From top to bottom: acceleration, velocity, z position, and x position. Every subject observed the same driving course.

The timing and degree of acceleration, deceleration and turns were all set once according to the constraints described above. The driving schedule was set prior to the experiment, and all of the subjects experienced the same driving schedule. The acceleration, velocity, z position, and x position of the simulated driving schedule are shown in Figure 3. The subjects experienced the same driving schedule under the three conditions described below, with about 30 minutes rest between sessions. When the entire experiment was over, the subjects were asked about the sameness of the driving course under the three conditions. None of the subjects noticed that the course was the same in each case.

**Figure 4:**
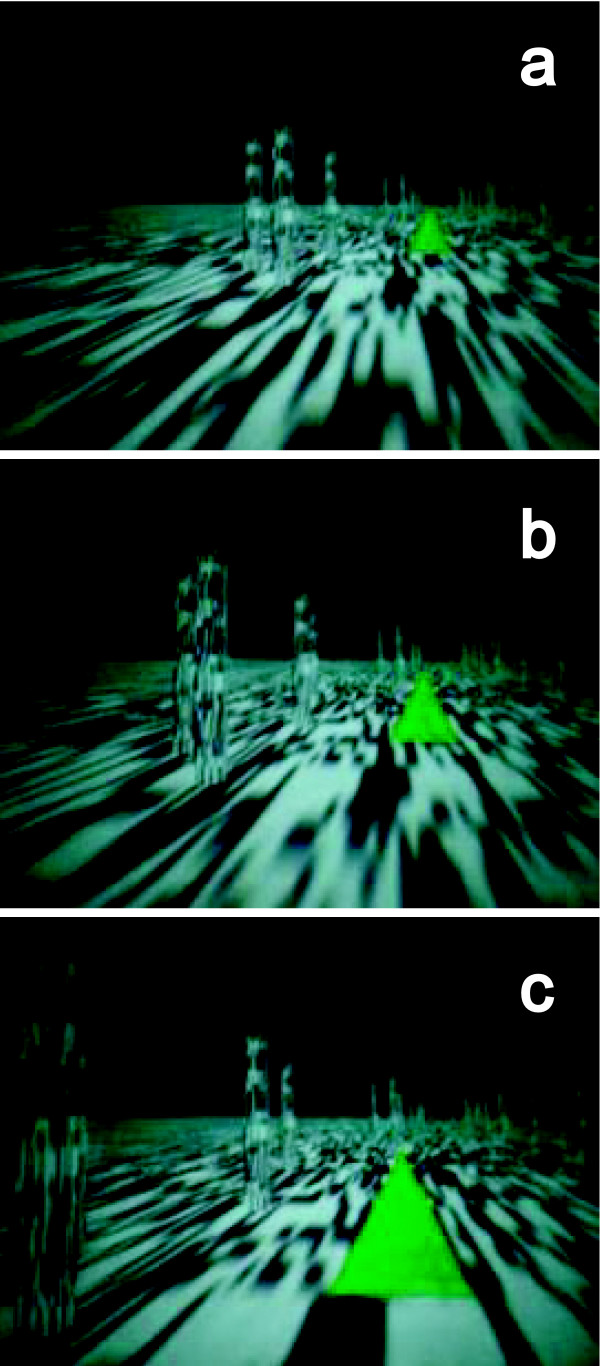
**Sample views with and without predictive signs**. Scattered objects showed the subjects their rough trajectory. Predictive signs appeared at the position of the acceleration (a), moved toward the subjects (b), and were visible until they collided with the subjects (c).

Four hundred rectangular parallelepipeds (0.25 × 0.25 × (0.5–2) m) were randomly arranged along the driving course as obstacles. They roughly, though not completely, informed observers of the driving course. The parallelepipeds were placed on either side of the invisible course, and the virtual automobile never collided with them. Random dot textures were mapped to the parallelepipeds and the world plane to emphasize depth perception. An example of what observers saw is shown in Figure 4.

#### Observation Conditions

The subjects experienced three observation conditions in random order: no signs, signs at 750-ms intervals, and signs at 3500-ms intervals. With no signs, observers sat in the chair locked on the motion base and experimenters presented both visual and vestibular information. Subjects observed the stimuli for approximately sixteen minutes. With the 750-ms and 3500-ms interval conditions, subjects observed the same stimuli as with no signs but were presented with signs that warned of acceleration or rotation 750 or 3500 ms before each event. The signs were triangles pointing up, down, right, and left for acceleration, deceleration, right rotation, and left rotation, respectively. Subjects were informed of the meaning of the signs before the experiments. The sides of the triangles were 50 cm long, and they appeared (center of mass) at 75 cm above the floor where events occurred. They moved toward observers and disappeared before colliding with observers (Figure 4). Subjects held a joystick and we asked them to report the direction of the triangles using with the joystick in the 750-ms and 3500-ms intervals and to report the direction of movement of the seat when no signs were present to motivate subjects to participate. After completion of all of the experiments, we explained the differences among the three sign conditions to the subjects and asked them which condition (no sign, 750-ms, or 3500-ms) was the most unpleasant.

#### Motion sickness questionnaire

At the end of each observation, we asked the subjects about whether they felt motion sickness. The questionnaire was based on the Graybiel score, a multi-symptom checklist for assessing motion sickness symptomatically [18]. It consisted of seven questions about nausea, sweating, salivation, level of consciousness, headaches, vertigo, and changes in complexion. The subjects rated their relative condition for each area on a scale of 0 – 5 (0 = none, 5 = strongly present). The total possible scores ranged from 0 to 50. Higher scores reflected more severe symptoms.

## Results

### Pre-experiment questionnaire on subject attributes, including motion sickness sensitivity

Prior to the experiment, all of the subjects filled out a questionnaire asking if they had a hangover, insufficient sleep, driving experience, tendency to car sickness, or medical conditions involving dizziness. None of the subjects complained of hangover, insufficient sleep or dizziness on the day of the experiments. None of the subjects had experienced dizziness that required medical attention. Concerning driving experience, four of the 21 subjects responded that they drove almost every day, seven reported driving occasionally and ten said they did not drive at all. Eight subjects reported no sensitivity to motion sickness, eight reported occasional sensitivity, none reported high sensitivity and four reported sensitivity in childhood. Driving experience and sensitivity to motion sickness results are listed by subject and by sex in Table 1.

**Table 1:** Subject attributes and the variation in LF/HF ratio

Name	Age	Sex	Driving frequency	Motion sickness sensitivity	No sign	750-Intv.	3500-Intv.
OTM	23	M	Everyday	Childhood	-	-	-
SSK	21	M	Occasionally	None	+	+	+
UEN	22	M	None	Childhood	+	+	-
HOK	28	M	Everyday	None	+	-	-
KWN	29	M	None	Occasionally	+	+	-
SUG	23	M	None	Childhood	+	-	+
JUK	21	M	Occasionally	Occasionally	+	-	-
KTM	24	M	None	None	+	+	+
MYS	28	M	None	Occasionally	-	-	+
NGW	27	M	Occasionally	None	+	-	-
SAT	28	M	Occasionally	None	+	-	+
SMY	23	M	None	None	+	+	-
STO	26	F	None	None	+	+	+
TCT	23	F	Occasionally	Occasionally	-	+	-
TJT	23	F	Everyday	Occasionally	-	+	+
UNO	25	F	None	Childhood	+	-	+
YMJ	21	F	Occasionally	Occasionally	+	-	+
YMS	24	F	None	Occasionally	+	-	+
YNG	23	F	Everyday	None	-	+	-
YSR	22	F	None	Occasionally	+	-	+
NSG	27	F	Occasionally	Occasionally	+	+	-

Name corresponds to the each titles of Figure 6.

Sex M = male, F = female

No sign, 750-Intv, and 3500-Intv + = Increase of LF/HF ratio, - = Decrease of LF/HF ratio from the first phase to the last phase.

### Self-assessment Graybiel score at the end of each session

In these experiments, the subjects were evaluated for motion sickness with seven items of the Graybiel score after each of the three sessions. The items were nausea, cold sweat, salivation, level of awareness, headache, dizziness, and pallor. During the experiments, one female subject reported 'severe' motion sickness immediately after the first observation (the session was ended immediately, so no Graybiel score could be given). That subject did not participate in the rest of the experiment. All of the other subjects completed the observation and almost none of them reported a feeling of motion sickness. Two of the 21 subjects reported the lowest degree of nausea and one subject reported the lowest degree of headache once in the second stage of the experiment. The observation conditions were different for each of those three subjects.

### Effect of observation conditions on overall activity of autonomic nervous system

**Figure 5:**
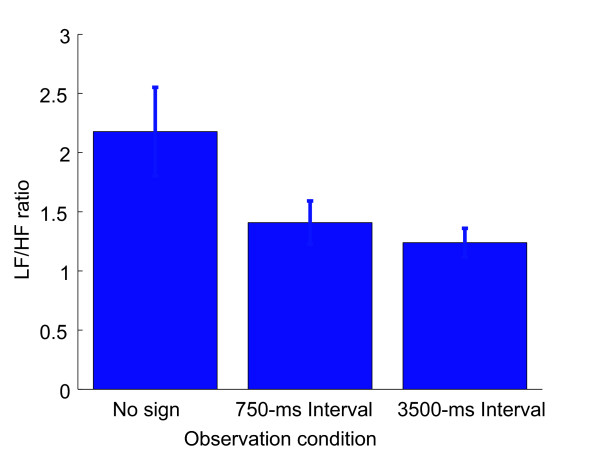
**Averaged LF/HF ratio of all subjects under three observation conditions**. Averaged LF/HF ratio from R-R interval of full observation period; error bar represents 1 SE.

We calculated the LF/HF ratio using the data for each trial (approximately sixteen minutes) of each observation condition. The average LF/HF ratio of all subjects under the three observation conditions is shown in Figure 5. The LF/HF ratio was the highest under the no-sign condition among the three observation conditions. A one-way within-group analysis of the variance (three observation conditions, ANOVA) was conducted on the LF/HF ratio. ANOVA revealed the main effect of the observation condition (*p *< 0.01) and the least significant difference (LSD) multiple comparison also revealed a significant difference between the no-sign condition and the 750-ms interval condition (*p *< 0.05), and the no-sign condition and the 3500-ms interval condition (*p *< 0.05).

### LF/HF ratio as a function of observation time

**Figure 6:**
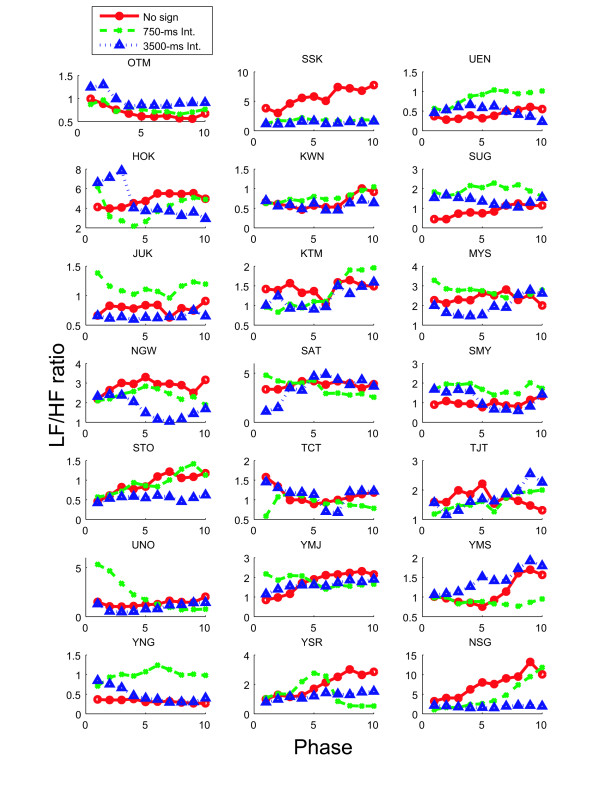
**Change of LF/HF ratio for individual subjects**. LF/HF ratio as a function of time under three conditions for each subject

In this section, we discuss the activity of the autonomic nervous systems of subjects and how it changed for each observation condition. We obtained approximately sixteen minutes of heart rate data for each observation condition and calculated the changing of the LF/HF ratio by moving the six-minute rectangular window. The change of the LF/HF ratio for each subject under the three observation conditions is shown in Figure 6, where each point plotted in the graphs shows the LH/HF ratio derived from the six-minute window (e. g. Phase 1 represented the LF/HF ratio from time = 0 to 6, Phase 2 represented the LF/HF ratio from time = 1 to 7, etc.). As a qualitative feature of the results, the no-sign condition often seemed to cause the LF/HF ratio to increase with time. The difference in the LF/HF ratio between phases 1 and 10 showed that sixteen of the twenty-one subjects had their LF/HF ratios increase with time under the no-sign condition, and ten had an increase under the 750-ms condition, and eleven had an increase under the 3500-ms condition (however the χ^2 ^test did not show a significant difference about the distribution of the positive/negative value of increments).

The subject information obtained prior to the experiments and the changes in the LF/HF ratios are presented in Table 1. The relations between change in the LF/HF ratios and subject sex, driving experience and motion sickness sensitivity are summarized in Tables 2, 3 and 4. Each table cell represents the number of subjects for whom the LF/HF ratio increased for the compared items (in Tables 3 and 4, the results for the subjects responding with "None" are compared with the results for subjects responding with either "Every day" or "Occasionally"). The results show that the percentages of LF/HF ratio increase were the highest under the 'No sign' condition for female subjects in Table 2 and the 'no driving experience' condition in Table 3. The LF/HF ratio of 90% for subjects with no driving experience is particularly striking (Table 3). The increase in the LF/HF ratio for persons who responded that they have not experienced motion sickness in questionnaire (Table 4) decreases in order of 'No sign', '750-Intv', and '3500-Intv' is also a very interesting result. However, the data in each cell is based on about 10 subjects at most, so the element of noise in the results must be considered, and the relationship between the subject attributes and the variation in LF/HF ratio should be further investigated with experimental data from a larger number of subjects.

**Table 2:** Gender and percentage of increase of LF/HF ratio

	No sign	750-Intv.	3500-Intv.
Male	0.83	0.42	0.42
Female	0.67	0.56	0.67

**Table 3:** Driving frequency and percentage of increase of LF/HF rati o

	No sign	750	3500
None	0.90	0.50	0.70
Yes	0.64	0.64	0.36

"Yes" including "everyday" and "occasionally" in Table 1.

**Table 4:** Motion sickness sensitivity and percentageo f increase of LF/HF ratio

	No sign	750-Intv.	3500-Intv.
None	0.88	0.63	0.50
Yes	0.77	0.31	0.46

"Yes" including "childhood" and "occasionally" in Table 1.

**Figure 7:**
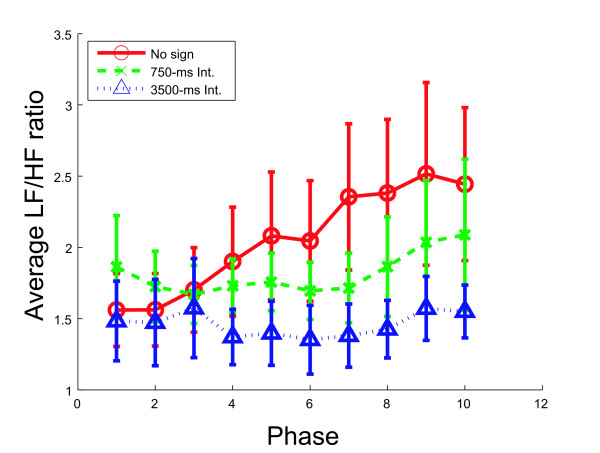
**Averaged LF/HF ratio change of all subjects**. Averaged data of Figure 6 for all subjects; error bar represents 1 SE.

The averaged LF/HF change data for each subject is shown in Figure 7. A two-way within-group analysis of variance (3 observation conditions × 10 phases, ANOVA) was conducted on the LF/HF ratio. The ANOVA revealed the main effect of phase(*p *< 0.001), but did not show the main effect of observation condition or the interaction between these two main effects. In a simple assessment of impressions conducted after the experiments, all of the subjects responded that the session with the 'No sign' condition was more unpleasant than the session with the 3500-ms condition. However eleven subjects reported after the experiment that they felt uncomfortable about the interval between the appearance of the signs and the acceleration under the 750-ms condition. We guessed that such a short interval causes subjects to be insufficiently ready for acceleration, and the accumulation of such a mental loading over time might have had a noise effect on the statistical analysis concerning the function of the predictive sign. Thus, as ad hoc analysis, we ignored the data of the 750-ms interval condition and conducted an ANOVA (2 observation conditions × 10 phases, ANOVA). The ANOVA revealed a significant tendency of the main effect of phase (*p *< 0.1) and observation condition (*p *< 0.1) and a significant interaction between observation and phase (*p *< 0.01). LSD multiple comparison also revealed a significant difference between the two observation conditions (no sign and 3500-ms interval conditions) at phases 4, 9 and 10 at *p *< 0.1, and 5, 6, 7, and 8 at *p *< 0.05. It also revealed a significant interaction between the no-sign condition and phase (*p *< 0.05) but not between the 3500-ms interval condition and phase.

## Discussion

Earlier research has pointed to the correlation between sensitivity to motion sickness and the LF/HF ratio [6,10]. Our results suggest that the presence of predictive signs affects the increase in the LF/HF ratio. The result that the LF/HF increases when no signs are presented is consistent with the previous results. On the other hand, however, a dissociation between subjective symptoms and the physiological response was seen, as no remarkable motion sickness was reported on the questionnaire. Much previous research has shown that the results of questionnaire surveys are not so sensitive to the motion sickness induced by mildly provocative VR content [19], and even when there is sensitivity, very low ratings result. The driving simulator we used in this research was designed with stimuli to prevent visual and vestibular conflict, so assuming that conscious sickness would not likely occur, we believe that slight psychological loads that do not produce serious illness could be detected by changes in heart rate within the range of our stimulus settings.

A previous study reported this kind of dissociation between physiological and psychological output in virtual environments [20]. Akiduki et al. presented a sensory conflict between visual and vestibular input to the subjects in which the rotation of the virtual environment around the vertical axis did not match the head movement of subjects. Their data suggested that the Graybiel scores for subjects changed significantly after twelve minutes by immersion in such a sensory conflict situation, while the amount of body sway area changed significantly after 20 minutes [20]. We could not compare our results with theirs directly because of differences in the active and passive experimental concern of the observers with the virtual environment. Our subjects received the visual and vestibular information passively while sitting in a driving simulator, while the subjects in Akiduki et al. [20] walked around the virtual space following guide lines and turned their heads freely. The sensory conflict in the virtual environment was a major difference between our study and theirs, and thus we cannot explain the disagreement between our results and theirs (physiological or psychological priority).

These results suggest that the activity of the sympathetic nervous system was greater under the no-sign condition than under the 3500-ms interval condition in the latter half of the observation period. The results, in other words, suggest that a long enough interval between the appearance of signs and acceleration maintained stable activity of the autonomic nervous systems of subjects. A short interval did not systematically change the activity of autonomic nervous system.

In our experimental set up, signs had no quantitative information about the acceleration, so they could have caused "false alarms" for the subjects when acceleration was small enough. Previous studies, moreover, suggested that some continuous mental tasks, such as the Stroop task, mirror drawing, and mental arithmetic [21,22] affect heart rate variability. Thus, the continuous interpretation of signs about acceleration in our experiment could increase the mental load on the subjects, even though the tasks are simple. Therefore, considering both the interval between signs and events and the gradual representation of events corresponding to the quantity of acceleration in the design of more effective signs, seems to be important.

## Conclusion

We reported on the effects of visual signs that informed subjects of acceleration on the activity of the sympathetic and parasympathetic nervous systems when the subjects observed a driving simulator that provided visual and vestibular information. Our results suggested that the effect of such signs on the stable activity of the autonomic nervous system depends on the timing of sign appearance. We pointed out the importance of the interval between sign and acceleration and the gradual representation of events corresponding to the quantity of acceleration.

## Authors' contributions

HW designed the study and participated in data collection. WT participated in data collection and helped with data analysis. HW drafted the manuscript. All authors helped with the interpretation of the results, reviewed the manuscript, and participated in the editing of the final version of the manuscript.
